# Neoadjuvant treatment in ovarian cancer: New perspectives, new challenges

**DOI:** 10.3389/fonc.2022.820128

**Published:** 2022-07-26

**Authors:** Adamantia Nikolaidi, Elena Fountzilas, Florentia Fostira, Amanda Psyrri, Helen Gogas, Christos Papadimitriou

**Affiliations:** ^1^ Oncology Department, Private General Maternity, Gynecological and Pediatric Clinic “MITERA“ Hospital, Athens, Greece; ^2^ Second Department of Medical Oncology, Euromedica General Clinic of Thessaloniki, Thessaloniki, Greece; ^3^ European University Cyprus, Engomi, Cyprus; ^4^ Molecular Diagnostics Laboratory, National Centre for Scientific Research ‘Demokritos’, Athens, Greece; ^5^ Section of Medical Oncology, Department of Internal Medicine, “Attikon” Hospital, National and Kapodistrian University of Athens School of Medicine, Athens, Greece; ^6^ First Department of Medicine, ‘Laiko’ General Hospital, National and Kapodistrian University of Athens School of Medicine, Athens, Greece; ^7^ Oncology Unit, Second Department of Surgery, “Aretaieion” University Hospital, National and Kapodistrian University of Athens School of Medicine, Athens, Greece

**Keywords:** ovarian cancer, NACT, bevacizumab, PARPi, immunotherapy

## Abstract

Ovarian cancer remains the leading cause of death from gynecological cancer. Survival is significantly related to the stage of the disease at diagnosis. Of quite importance is primary cytoreductive surgery, having as a goal to remove all visible tumor tissue, and is the standard primary treatment in combination with platinum-based chemotherapy for patients with advanced ovarian carcinoma.

Neo-adjuvant chemotherapy (NACT) has been implemented mostly in treating advanced disease, with studies performed having numerous limitations. Data extrapolated from these studies have not shown inferiority survival of NACT, compared to primary debulking surgery. The role of NACT is of particular interest because of the intrinsic mechanisms that are involved in the process, which can be proven as therapeutic approaches with enormous potential. NACT increases immune infiltration and programmed death ligand-1 (PDL-1) expression, induces local immune activation, and can potentiate the immunogenicity of immune-exclude high grade serous ovarian tumors, while the combination of NACT with bevacizumab, PARP inhibitors or immunotherapy remains to be evaluated. This article summarizes all available data on studies implementing NACT in the treatment of ovarian cancer, focusing on clinical outcomes and study limitations. High mortality rates observed among ovarian cancer patients necessitates the identification of more effective treatments, along with biomarkers that will aid treatment individualization.

## Introduction

Ovarian cancer (we also include tubal and peritoneal cancer in the term ovarian cancer) is the third most common gynecological cancer after cervical and endometrial cancer (1) and the leading cause of death from gynecological cancer in developed countries (2). Although the incidence of ovarian cancer is significantly lower compared to breast cancer, ovarian cancer is three times more deadly and its mortality is expected to increase significantly by 2040 due to lack of accurate screening methods for early diagnosis (3, 4).

Approximately 90% of ovarian cancers are of epithelial origin and most of them have serous histology. The overall survival (OS) of patients with ovarian cancer is related to the stage of disease at diagnosis. More than 75% of patients have already advanced disease at diagnosis, either stage IIIC or IV, leading to poor clinical outcomes. Primary cytoreductive/debulking surgery (PDS) with the goal to remove all visible tumor tissue, is the standard primary treatment for patients with advanced ovarian carcinoma, followed by platinum-based chemotherapy. Observational studies report that the achievement of optimal debulking with residual disease <1 cm, is associated with increased OS (5).

Importantly, interval debulking surgery (IDS) is not associated with improved prognosis in patients with residual disease after PDS or in cases where PDS is performed by a non-specialist surgeon and, therefore, is not indicated for these patients (6, 7). It is accepted that surgery for ovarian cancer should be performed by specialist gynecological oncologists in high volume centers.

IDS following neoadjuvant chemotherapy (NACT) comprising more commonly of three cycles of chemotherapy, is an alternative treatment option for patients who are unable to undergo primary complete resection.

## NACT clinical trials

Two retrospective meta-analyses compared NACT and interval debulking to primary cytoreduction and adjuvant chemotherapy. The first one comprised of twenty-one studies, including a total of 835 patients (8). This trial convincingly showed that the main parameters associated with patient survival were the use of platinum-based regimens and the performance of optimal debulking surgery. The above meta-analysis also reported that the weighted average median survival of patients subjected to NACT was 24.5 months. The data also suggested that the performance of maximal interval cytoreductive surgery is a significant predictor of median survival time. However, the increase in the number of NACT cycles was associated with worse OS (8). Another meta-analysis of twenty-one studies showed that NACT indeed increased the rate of optimal cytoreduction, despite unfavorable conditions. This increase, however, did not have a favorable impact on improved OS, especially as compared with primary debulking surgery in patients with low-risk disease (9).

Studies evaluating the use of NACT in advanced ovarian cancer have several limitations. Primarily, PDS needs to be performed by a gynaecologic oncologist, with maximal surgical effort, as demonstrated by the GOG-152 trial (10). Secondly, the aforementioned studies included patients from different centres, treated by surgeons with diverse surgical experience, which can be reflected in differences in operation time. Foremost, the most important limitation of the two aforementioned meta-analyses is the significant heterogeneity between the included studies.

The first trial implementing NACT in advanced ovarian cancer, the EORTC 55971 trial (11), randomized 632 patients to receive at least six cycles of platinum-based chemotherapy after primary cytoreductive surgery or three cycles of neoadjuvant platinum-based chemotherapy followed by interval debulking in patients with objective response or stable disease, followed by another three cycles of platinum-based chemotherapy. In the intention- to-treat population, median OS was similar in both groups of patients (29 months in the primary surgery group vs. 30 months in the NACT group, HR=0.98, 90% confidence interval [CI], 0.84 to 1.13; p=0.01), as was progression-free survival (PFS), i.e., 12 months in both groups. The strongest independent predictive factors of improved OS were the absence of residual disease after surgery, stage IIIC disease, small tumour size at randomization, endometrioid histology and younger age at diagnosis.

Of note, the study included patients with extensive disease, a parameter that complicates the completion of R0 resection, the main independent prognostic factor in advanced ovarian cancer. The study also highlighted the diverse outcomes of cytoreductive surgery among different countries.

EORTC 55971 trial confirmed that the extent of residual disease, either after PDS or IDS, is an important prognostic factor (11). Additionally, NACT was not shown to improve OS, postoperative mortality, or overall mortality, reduce the rate of adverse events or improve quality of life. Sub-analyses demonstrated that patients with stage IIIC disease and diameter of the largest neoplastic lesion <45 mm had improved OS after primary cytoreduction compared to IDS, while patients with stage IV disease and metastatic lesions measuring> 45 mm had improved survival after NACT and IDS *vs* primary cytoreduction.

The CHORUS trial, another phase III, non-inferiority study, randomized 550 women with advanced ovarian cancer and poor performance status to either primary cytoreduction followed by adjuvant chemotherapy (276 patients) or to NACT followed by IDS (274 patients) (12). The study demonstrated that OS after NACT and IDS was not inferior to that in patients receiving primary surgery and adjuvant chemotherapy. Specifically, median OS was 23.7 months in the IDS group *vs* 25.8 months in NACT group (HR=0.89; 95% CI 0.73-1.08), while PFS was 12 months in the NACT group *vs* 10.7 months in the primary surgery group (HR=0.91; 95%CI 0.76-1.09). In addition, the study showed that the administration of NACT followed by IDS led to a statistically significant decrease in postoperative complications of Grade 3 and 4. CHORUS was the second trial to investigate the timing of surgery in newly diagnosed advanced ovarian cancers (12). Compared to similar trials, patients were older (median age 65 years) and had a poor performance status (only 30% of patients had WHO performance status 0) (11, 12).

EORTC 57971 and CHORUS trials, also demonstrated that before selecting patients for NACT, it is important to rule out other primary tumours, especially those of gastrointestinal origin. A CA-125 to CEA ratio higher than 25, has been shown to be a useful tool for ruling out primary gastrointestinal tumours with metastases to the peritoneum or ovaries. Limitations of these two trials include low complete cytoreduction rates in the PDS arm and low accrual rates in selected centres.

A pooled analysis of individual patient data from CHORUS and EORTC 55971 trials focused on long-term outcomes of patients and the determination of preferable therapeutic decisions for subgroup populations (13). Overall, data from 1,220 patients were included in the analysis, while the median follow-up time was 7.6 months. This pooled analysis showed that PDS remains the gold-standard for women with FIGO stage ≤IIIB. On the contrary, NACT should be the standard-of-care for most patients with stage IV ovarian cancer. In patients with FIGO stage IV disease, primary cytoreduction should only be considered on an individual basis and in exceptional circumstances. Finally, patients with FIGO stage IIIC disease with extra pelvic metastases <5 cm, should be considered for PDS, since these patients were shown to have significantly improved PFS with upfront cytoreduction.

In the Japanese JCOG0602 phase III trial, 149 patients were randomized to primary cytoreduction and 152 to NACT followed by IDS (14). The aim of the study was to assess whether the efficacy of NACT would be non-inferior to PDS and whether it would be associated with reduced surgical invasiveness, and therefore a decrease in adverse events. Patients were randomized without undergoing diagnostic surgery prior to treatment, in contrast to the EORTC 55971 and CHORUS trials, where diagnostic laparotomy or laparoscopy had preceded (in 34.5% of patients randomized to PDS and in 38.3% of patients randomized to the NACT in EORTC 55971 trial, and in 16% of patients randomized to NACT in CHORUS). This study demonstrated that NACT was associated with lower level of invasiveness of interval debulking surgery, leading to lower frequency of postoperative adverse events and blood/albumin transfusions, lower frequency of abdominal organ and distant metastases resection and shorter total operation time. Finally, median OS was 49.0 and 44.3 months in the PDS and NACT group (HR=1.052), respectively, while median PFS was 15.1 and 16.4 months in the PDS and NACT (15).

The Italian SCORPION-NCT01461850, open-label, phase III trial (16, 17) enrolled 171 patients that were randomized to receive either NACT and subsequent interval debulking surgery or adjuvant chemotherapy after PDS. This study was designed to overcome the limitations of the previous EORTC 55971 and CHORUS trials. The most important characteristic of the SCORPION trial was the inclusion of a single institution (and its affiliates) with high accrual rates of patients per year and commitment to maximal surgical effort. IDS was associated with lower post-operative complication rates, including post-operative deaths. This trial, although underpowered to detect a difference, also showed that NACT and IDS had similar efficacy to primary cytoreduction and adjuvant chemotherapy. However, higher rates of complete resection were achieved after NACT (R0 was achieved in 47.6% of patients in the primary debulking arm vs 67.0% in the interval debulking arm, *p*=0.0001). The toxicity profile also differed between the two treatment arms, with significantly fewer postoperative complications in the arm of NACT. Furthermore, there was no difference in median PFS (15 months in the PDS arm *vs* 14 months in the IDS arm, HR=1.05) or median OS (41 months *vs* 43 months, respectively, HR=1.12). Finally, the prolonged median OS in the SCORPION trial (43 months for patients assigned to NACT) compared to the median OS in the individual patient meta-analysis of the EORTC and CHORUS trials (27 months) may reflect the characteristics of patient population, including younger age and better performance status.

Concerning data on Quality of Life (QoL), the SCORPION trial found a statistical improvement in six different scales in QoL scores in the NACT arm, compared to PDS arm (15). On the contrary, the EORTC 55971 and CHORUS trials found no difference in the QoL scores between the two groups of patients (11, 12). **Table 1** summarizes all randomized phase III trials that compared NACT and IDS with PDS followed by adjuvant chemotherapy in patients of advanced epithelial ovarian cancer (EOC).

**Table 1:** Randomized phase III trials that compared neoadjuvant chemotherapy and interval debulking surgery with primary debulking surgery in patients of advanced epithelial ovarian cancer.

Study	Population	No of Patients	Interventions	Complete Resection Rate (%)		PFS^a^ (months)	OS^b^(months)	Median Operative Time (min)	Peri- and postoperative AEs^c^ in PDS^d^ and NACT^e^-IDS^f^
EORTC Van der Burg et al, 1995 (7)	Patients with FIGO stage IIB-IV EOC^g^ who had residual lesions >1 cm after PDS	138 140	*Control Arm* 6 of Cyclophosphamide and Cisplatin *Experimental Arm* 3 cycles of Cyclophosphamide and Cisplatin followed by IDS. Postoperatively, another 3 cycles of Cyclophosphamide and Cisplatin.	NA 17		13 18 *p *= 0.013	20 20 *p = *0.012	NA^h^ NR^i^	NR
EORTC 55971 Vergote et al, 2010 (12)	Patients with FIGO stage IIIC-IV EOC, fallopian-tube carcinoma, or primary peritoneal carcinoma	336 333	*PDS Arm* At least six courses of Platinum-based chemotherapy *NACT Arm* 3 courses of neoadjuvant Platinum-based chemotherapy followed by IDS in patients with a response or stable disease, followed in turn by at least 3 courses of adjuvant Platinum-based chemotherapy	19.4 51.2		12 12	29 30	165 (312 when R0) 180 (194 when R0)	In the PDS arm postoperative death 2.5% versus 0.7% in NACT-IDS; hemorrhage 7.4% versus 4.1%; infection 8.1% versus 1.7%
CHORUS Kehoe et al, 2015 (13)	Patients with FIGO stage III-IV EOC, fallopian-tube carcinoma, or primary peritoneal carcinoma	276 274	*PDS Group* 6 cycles of Carboplatin AUC 5 or 6 plus Paclitaxel 175 mg/m^2^ every 3 weeks; an alternative Carboplatin combination regimen; or Carboplatin monotherapy *NACT Group* 3 cycles of primary chemotherapy, then IDS, followed by 3 more cycles of chemotherapy	17 39	10.7 12 HR = 0.91		22.6 24.1 HR = 0.87	120 120	PDS group had more Grade 3 or 4 AEs (24%) than the NACT-IDS group (14%) *p* = 0.007; peri-operative death 6% versus <1% (*p = *0.001)
JCOG0602 Onda et al, 2016 and 2020 (15, 16)	Patients with FIGO stage III-IV EOC, fallopian-tube carcinoma, or primary peritoneal carcinoma	149 152	*PDS Arm* 8 cycles of Paclitaxel 175 mg/m^2^ and Carboplatin AUC 6 *NACT Arm* 4 cycles of NACT; then IDS (unless disease progression was noted) followed by 4 cycles of postoperative chemotherapy	12 31	15.1 16.4 HR = 0.96		49 44.3 HR = 1.052	302 240 *p*<0.001	Grade 3 or 4 AEs after surgery were 15% in PDS arm versus 4.6% in NACT-IDS (*p* = 0.005)
SCORPION trial Fagotti et al, 2016 and 2020 (17, 18)	Patients with stage IIIC-IV EOC and PI^h^ score ≥ 8 or ≤ 12 (considered high tumor load) with no evidence of mesenteric retraction	84 74	*PDS (Arm A)* 6 cycles of Paclitaxel 175 mg/m^2^ and Carboplatin AUC 5 *NACT-IDS (Arm B)* 3-4 cycles of NACT; then IDS (unless disease progression was noted); chemotherapy was resumed within 4 weeks after IDS in order to complete the 6 planned cycles	47.6 77 *p = *0.001	15 14 HR = 1.05		41 43 HR = 1.12	460.6 253.2 *p*<0.0001	Major complications in 46.4% of patients in the PDS arm versus 9.5% in NACT-IDS (*p* < 0.0001)

a Progression-free survival.

b overall survival.

c adverse events.

d primary debulking surgery.

e neoadjyvant chemotherapy.

f interval debulking surgery.

g epithelial ovarian cancer.

h laparoscopic predictive index.

TRUST is an ongoing international, randomized, controlled multi-centre trial, investigating OS after primary cytoreductive surgery versus NACT and subsequent IDS in patients with FIGO stage IIIB-IVB ovarian carcinoma (18). To ensure adequate surgical quality, participating centres needed to fulfil specific quality assurance criteria (e.g., ≥50% complete resection rate in upfront surgery for FIGO IIIB-IVB patients and ≥36 debulking surgeries/year) and allow independent audits by TRUST quality committee delegates. Patients in the PDS arm underwent surgery followed by six cycles of platinum-based chemotherapy, whereas patients in IDS arm received three cycles of NACT after histologic confirmation of the disease, followed by IDS and subsequently, another three cycles of platinum-based chemotherapy. Patient recruitment was completed in approximately mid-2019 and the results are expected after a 5 year-follow-up in 2024.

Another study protocol was recently published, the SGOG SUNNY (SOC-2) (19), a randomized, open-label, multicentre, phase III clinical trial in Asian countries for patients with FIGO stages IIIC or IV ovarian cancer of any tumor burden. SUNNY trial is investigating OS with quality guarantee, PFS, quality of life (QoL), and treatment-free intervals (TFIs) after PDS vs NACT-IDS. The aim of this trial is to further investigate the role of NACT-IDS and PDS in treatment of advanced EOC using a well-designed protocol with surgical quality assurance. For this reason, all participating centres needed to be specialised ovarian cancer centres with multidisciplinary approach. In addition, maximal cytoreduction, even in patients with high tumor burden, needed to be conducted either in interval or upfront surgery. Additionally, the percentage of patients with no gross residual (NGR) disease needed to be at least 50% in the PDS group. Survival data are expected in a few years.

Although clinical data support the use of NACT, there is still no consensus on the number of NACT cycles. In fact, in daily clinical practice, patients receive 2 to 6 cycles of NACT before surgery according to the physicians’ choice. Colombo et al. (20) and Xu et al. (21), reported that more than four cycles of NACT have a negative effect on patients’ outcomes. Bogani et al. (22) retrospectively reviewed the data of consecutive patients undergoing NACT and PDS in four Italian centers and evaluated the survival outcomes. Although most patients received 3 or 4 cycles, approximately 25% of patients had five or more cycles. The investigators concluded that the number of cycles had no effect on the ability to achieve complete and optimal cytoreduction. α trend toward worse OS was observed for patients with residual disease at IDS and patients receiving at least 4 cycles (HR=1.76; 95% CI, 0.95-3.22; *p*=0.06). In this context, the results of two ongoing phase III randomized trials are eagerly awaited. Both GOGER-01 (23) and CHRONO (24) trial, randomize patients with phase III-IV EOC, fallopian tube carcinoma or primary peritoneal carcinoma to receive 3 or 6 cycles of NACT with paclitaxel and carboplatin. The primary endpoint of GOGER-01 is the percentage of patients who obtain a complete cytoreduction at surgery (R0 rate), whereas that of CHRONO the disease-free survival (DFS). Selected ongoing phase II and III clinical trials investigating neoadjuvant chemotherapy in EOC are shown in **Table 2**.

**Table 2:** Selected current phase II and III clinical trials investigating neoadjuvant chemotherapy in epithelial ovarian cancer.

Study Identifier	Type of Study	Study Population	Primary endpoint (s)	Regimen/Treatment Arms
NCT04885270	Phase II	Patients with ovarian, primary peritoneal or fallopian tube cancer (FIGO stage IIIC-IV)	Optimal debulking rate	2-6 cycles of NACT^a^ with Paclitaxel 135 mg/m^2^ IV + Cisplatin 75 mg/m^2^ (q 3 weeks) followed by IDS^b^ and another 6 cycles of adjuvant chemotherapy
NCT04606914	Phase II	Patients with high grade serous epithelial ovarian cancer (FIGO stage III-IV)	PFS^c^ ORR^d^	NAT with 4 cycles of Carboplatin AUC 5 (q 3 weeks) plus 3 cycles of Mirvetuximab^e^ 6 mg/kg (starting with cycle #2 of Carboplatin) followed by IDS and then by another 3 cycles of adjuvant chemotherapy
NCT03448354	Non-randomized with parallel assignment	Patients with ovarian, primary peritoneal or fallopian tube cancer	PFS	*Non Intervention Arm* 3 cycles of NACT followed by IDS *Experimental Arm* 3 cycles of NACT followed by IDS and HIPEC^f^ (Paclitaxel 175 mg/m^2^, 90 min) in case of residual disease <5 mm
NCT02125513 GOGER-01	Randomized Phase II	Patients with ovarian, primary peritoneal or fallopian tube cancer (FIGO stage IIIC-IV)	R0 resection rate	*Arm A (Comparator)* 3 cycles of ΝΑCΤ with Carboplatin AUC 5 and Paclitaxel 175 mg/m^2^ (q 3 weeks), followed by IDS. A weekly Paclitaxel schedule is allowed while Bevacizumab use is optional. *Arm B (Experimental)* 6 cycles of NACT with 6 Carboplatin AUC 5 and Paclitaxel 175 mg/m^2^ (q 3 weeks), followed by IDS. A weekly Paclitaxel schedule is allowed while Bevacizumab use is optional.
NCT03579394 CHRONO	Randomized Phase II	Patients with ovarian, primary peritoneal or fallopian tube cancer (FIGO stage IIIB-IVA)	DFS^g^	*Arm A (Comparator)* 3 cycles of NACT with Carboplatin AUC 5-6 and Paclitaxel 175 mg/m^2^ (q 3 weeks), followed by IDS, then followed by 5 more cycles of Carboplatin and Paclitaxel chemotherapy plus Bevacizumab (for a total of 15 months). Alternatively, weekly Paclitaxel/Carboplatin schedules can be used. *Arm B (Experimental)* 6 cycles of NACT with Carboplatin AUC 5 and Paclitaxel 175 mg/m^2^ (q 3 weeks), followed by IDS, then followed by 2 more cycles of Carboplatin and Paclitaxel chemotherapy plus Bevacizumab (for a total of 15 months). Alternatively, weekly Paclitaxel/Carboplatin schedules can be used.
NCT02859038 SUNNY	Phase III	Patients with ovarian, primary peritoneal or fallopian tube cancer (FIGO stage IIIC-IV)	OS^h^	*Arm A (Comparator)* 3 cycles of Paclitaxel 175 mg/m^2^ or Docetaxel 60-75 mg/m^2^ plus Carboplatin AUC 5 followed by IDS with a maximal cytoreduction of complete gross resection, then followed by another 3 cycles of chemotherapy *Arm B (Experimental)* Upfront PDS^i^ with a maximum cytoreduction followed by 6 cycles of adjuvant Paclitaxel 175 mg/m^2^ or Docetaxel 60-75 mg/m^2^ plus Carboplatin AUC 5
NCT04515602 FOCUS	Phase III	Patients with ovarian, primary peritoneal or fallopian tube cancer (FIGO stage IIIC-IV)	OS	*Arm A (Comparator)* 3 cycles of Paclitaxel 175 mg/m^2^ or Docetaxel 60-75 mg/m^2^ plus Carboplatin AUC 5 followed by IDS with a maximal cytoreduction of complete gross resection, then followed by another 3 cycles of chemotherapy and maintenance with a PARPi for patients with *BRCA* mutation and CR/PR after first-line chemotherapy *Arm B (Experimental)* Upfront PDS with a maximum cytoreduction followed by 6 cycles of adjuvant Paclitaxel 175 mg/m^2^ or Docetaxel 60-75 mg/m^2^ plus Carboplatin AUC 5 and maintenance with a PARPi for patients with *BRCA* mutation and CR/PR after first-line chemotherapy
NCT04575935	Phase III	Patients with high grade ovarian, primary peritoneal or fallopian tube cancer (FIGO stage IIIC-IV)	DFS	*Comparator Arm* 3-4 cycles of standard of care NACT, followed by laparotomy, then followed by standard of care adjuvant chemotherapy *Experimental Arm* 3-4 cycles of standard of care NACT, followed by minimally-invasive surgery (MIS), then followed by standard of care adjuvant chemotherapy.

a Neoadjuvant chemotherapy.

b interval debulking surgery.

c progression free survival.

d objective response rate.

e a high affinity humanized monoclonal antibody against folate receptor α (FRα) that is conjugated to a cytotoxic maytansinoid.

f hyperthermic intraperitoneal chemotherapy.

g disease free survival.

h overall survibal.

i primary debulking surgery.

Bartles et al. (25), published a meta-analysis intended to review the morbidity and mortality associated with PDS in comparison to IDS. Overall, seventeen studies comprising 3,759 patients were selected, among which four randomized trials. The results demonstrated that mortality and morbidity rates were significantly lower with NACT as compared to PDS. Specifically, NACT was related to significantly lower perioperative morbidity and 30-day post-operative mortality, as well as increased complete cytoreduction rates, compared to PDS, while it did not offer an OS benefit.

## The role of HIPEC

Another controversial issue in the therapeutic era of advanced ovarian cancer is the use of hyperthermic intraperitoneal chemotherapy (HIPEC) after NACT. The benefit from HIPEC is based on non-randomized clinical trials or data from retrospective studies. Spiliotis et al. demonstrated improved OS using HIPEC in patients with recurrent EOC after CRS and then followed by systemic chemotherapy compared to CRS and systemic chemotherapy alone (26) However, the randomization process was not described in detail, and primary end points were not clearly defined. In a multicenter phase III clinical trial patients were randomized after 3 cycles of neoadjuvant chemotherapy to undergo interval debulking with or without HIPEC (27). Randomization was performed at the time of surgery in cases where surgery would result in complete or at least optimal cytoreduction. The addition of HIPEC to complete or optimal interval cytoreductive surgery resulted in longer median recurrence-free survival, by 3.5 months, and longer median OS, by 11.8 months, than surgery alone.

The OV21/PETROC, was a phase II trial, that evaluated the use of intraperitoneal chemotherapy in patients who received 3-4 cycles of NACT followed by IDS and optimal cytoreduction (28). Three different postoperative regimens were assessed, including (a) intravenous (IV) carboplatin/paclitaxel, (b) intraperitoneal (IP) carboplatin with IV/IP paclitaxel and (c) IP cisplatin with IV/IP paclitaxel regimen. No difference in PFS or OS was observed among the three groups of patients (28). In OVHIPEC, an open-label randomized phase 3 trial, patients with stage III ovarian cancer, who were not eligible for PDS based on the extent of their disease, were randomized 1:1 to receive IDS with or without HIPEC after 3 cycles of NACT with carboplatin and paclitaxel (27). The addition of HIPEC was associated with improved OS (45.7 months for the HIPEC group vs. 33.9 months for IDS-only group, hazard ratio, 0.67; 95% CI, 0.48 to 0.94; p=0.02) (27). In this study questions arose about potential imbalances in critical prognostic variables, including histological subtype, FIGO substage, BRCA status, response to neoadjuvant chemotherapy, and hospital size (29). Therefore, the results of this trial need to be interpreted with caution. However, two main considerations would be considered in trial with HIPEC: study design addressing in only a small population of patients and the heterogenity of results between the various centers.

In summary, NACT is indicated for the treatment of patients with FIGO stage IV as well as for patients with FIGO stage III when optimal debulking cannot be achieved or when upfront surgery is contraindicated due to comorbidities.

## Failure of NACT

Despite high response rates observed in patients receiving NACT, selected patients with advanced ovarian cancer have been shown to progress during or after NACT. NACT has been associated with platinum resistance, possibly through the following mechanisms: 1) difficulty in detecting residual cancer cells during IDS, 2) enhancement of stemness of ovarian cancer cells and 3) induction of gene mutations that promote resistance in platinum. IDS is not a viable option for non-responding to NACT patients. These patients, with unfavorable prognosis, would preferably be treated as platinum-resistant (30).

## The role of bevacizumab

The addition of bevacizumab to adjuvant chemotherapy followed by maintenance bevacizumab monotherapy after primary cytoreduction in stage IIIB-IV ovarian cancer has been associated with benefit in PFS, but had no significant impact on OS according to the initial analyses of two randomized phase III trials published in 2011 (GOG218 and ICON7) (31, 32).

A single-institution case-control study compared toxicity, perioperative outcomes of interval debulking and PFS in a series of patients with high grade serous advanced ovarian cancer, who received NACT with or without bevacizumab (33). Investigators showed that careful consideration prior to attempting bevacizumab based NACT in women with diffuse bowel involvement at laparoscopic evaluation is warranted. Nevertheless, the incorporation of bevacizumab into NACT prolongs PFS (18 months in the NACT-bevacizumab arm *vs* 10 months in the NACT arm, *p*=0.001) without affecting the safety of IDS.

GEICO 1205/NOVA, a phase II randomized, open, multicentre trial explored NACT with or without bevacizumab in patients with FIGO stage III-IV ovarian cancer and ECOG performance status 0-2 (34, 35). Surgical feasibility (rate of patients for whom surgery was feasible) was increased in the bevacizumab group (88.6% vs 66.7%, *p*=0.029), while there was no difference in optimal surgery rate (63.6% vs 65.7%, *p*=0.858) and median PFS (20.3 months in both arms).

In a subgroup analysis of the MITO16A-MaNGO OV2A phase IV trial, seventy-nine patients underwent interval debulking surgery after neo-adjuvant paclitaxel, carboplatin and bevacizumab (36). The median number of chemotherapy and bevacizumab cycles before interval debulking surgery was three. Although the proportion of stage IV patients included in this sub-analysis was higher than in other studies, 86.5% of them had residual tumours <1 cm. The rate of postoperative complications was similar to what expected without bevacizumab. Finally, the multicentre, open-label, non-comparative phase II ANTHALYA study, randomized patients in a 2:1 to receive four 4 of NACT (paclitaxel 175mg/m^2^ and carboplatin 5 AUC, every three weeks) with or without bevacizumab (37). NACT with bevacizumab achieved high complete resection rate in patients who underwent IDS (85.5%). Although in patients with initially unresectable FIGO stage IIIC/IV ovarian, tubal, or peritoneal adenocarcinoma, the complete resection rate with the addition of bevacizumab was significantly higher than with chemotherapy alone, the role of bevacizumab in this setting should be further investigated. The results of two ongoing prospective trials are awaited. In conclusion, data show that the addition of bevacizumab to NACT is safe, however, its efficacy remains under evaluation. **Table 3** summarizes the previously presented studies with bevacizumab in the neoadjuvant setting.

**Table 3:** Studies that evaluated the incorporation of bevacizumab into the neoadjuvant treatment of advanced epithelial ovarian cancer.

Study	Type of Study	Population	No of Patients	NACT^b^	Complete Resection Rate (%)	PFS^c^ (months)	Toxicity of IDS^d^
Petrillo et all, 2015 (29)	Single-institutional case-control study	Unresectable high-grade serous advanced EOC^a^	75	*Control Arm* 3-6 cycles of Carboplatin AUC 5 and Paclitaxel 175 mg/m^2^, every 3 weeks *Investigational Arm* 3-6 cycles of Carboplatin AUC 5 and Paclitaxel 175 mg/m^2^ plus Bevacizumab 15 mg/kg, every 3 weeks	72.3 80.0	10 18 *p* = 0.001	One death (5%) due to perforation
GEICO1205/NOVA Garcia Garcia et al, 2017 (30)	Randomized Phase II open label multicenter study	Patients with high grade serous or endometrioid EOC, FIGO stage III-IV, ECOG 0-2, considered unresectable and in whom NACT and IDS were planned	71	*Control Arm* 4 cycles of Carboplatin AUC 6 and Paclitaxel 175 mg/m^2^, every 3 weeks *Experimental Arm* 4 cycles of Carboplatin AUC 6 and Paclitaxel 175 mg/m^2^ with at least 3 courses of Bevacizumab 15 mg/kg, every 3 weeks	63.6 65.7	20.13 20.36	69.7% Grade 3-4 AEs^e^ in Control Arm versus 42.9% in Bevacizumab Arm (*p *= 0.026)
ANTHALYA Rouzier et al, 2017 (33)	Randomized multicenter, open-label, non-comparative phase II study	Patients with initially unresectable FIGO stage IIIC/IV ovarian, tubal or peritoneal adenocarcinoma.	95	*Control Arm* 4 cycles of Carboplatin AUC 5 and Paclitaxel 175 mg/m^2^, every 3 weeks *Experimental Arm* 4 cycles of Carboplatin AUC 6 and Paclitaxel 175 mg/m^2^ with 3 courses of Bevacizumab 15 mg/kg, every 3 weeks. Postoperatively, all patients received the same chemotherapy regimen (cycles 5-8) and Bevacizumab (cycles 6-26)	58.6 51.4	NR^f^ NR	63% Grade 3-4 AEs in Control Arm versus 62% in Bevacizumab Arm
MITO-16A-MaNGO OV2A Daniele et al, 2017 (32)	Phase IV	Subgroup analysis of FIGO stage IIIB- IV patients who received NACT followed by IDS	79	3-4 cycles of Carboplatin AUC 5, Paclitaxel 175 mg/m^2^ plus Bevacizumab 15 mg/kg, every 3 weeks. Postoperatively, all patients received the same regimen followed by Bevacizumab maintenance for up to 16 cycles	63.5	NR	The rate of perioperative complications was similar to what expected without Bevacizumab
Komiyama et al, 2018 (34)	Single‐center feasibility study	Patients with stage IIIC-IVA EOC, fallopian tube cancer, or primary peritoneal cancer in whose optimal surgery is unlikely to be achieved according to exploratory laparotomy	33	4 cycles of Carboplatin AUC 5 and Paclitaxel 175 mg/m2 plus 3 courses of Bevacizumab 15 mg/kg, every 3 weeks. Postoperatively, all patients received another 4 cycles of chemotherapy plus Bevacizumab (cycles 5-22)	60.9	NR	No complications ≥ Grade 3

a Epithelial ovarian cancer.

b neoadjuvant chemotherapy.

c progression-free survival.

d interval debulking surgery.

e adverse events.

f not reported.

## The role of PARP inhibitors

Four phase III trials evaluated PARP inhibitors in the setting of front-line treatment of ovarian cancer, namely SOLO-1, PAOLA-1/ENGOT-OV25, PRIMA/ENGOT-OV26 and VELIA/GOG-3005 (38–42).

SOLO-1 trial (40) compared maintenance treatment with olaparib (for up to 2 years in patients with no evidence of disease, or beyond in case of partial response at 2 years) *vs* placebo in patients with newly diagnosed advanced ovarian cancer with a *BRCA1* and/or *BRCA2* mutation (39). In this trial, ninety-four patients (36%) in the olaparib arm and forty-three patients (33%) in the placebo arm received NACT (40).

PAOLA-1/ENGOT-OV25- trial (41) investigated the co-administration of bevacizumab and olaparib as maintenance treatment *vs* bevacizumab treatment alone. Olaparib was administered for up to 2 years (or beyond in patients with partial response at 2 years) but bevacizumab was discontinued after 15 months of treatment. Overall, 228 patients (42%) in the olaparib/bevacizumab arm and 110 patients (40%) in the placebo/bevacizumab arm received NACT. Among patients who underwent IDS, no macroscopic residual disease was found in 71% of them in the olaparib/bevacizumab arm and in 68% in the placebo/bevacizumab arm. Median PFS in patients who underwent IDS and had no residual disease, was 22.1 months in the olaparib/bevacizumab arm and 17.7 months in the placebo/bevacizumab arm (HR=0.61, 95% CI 0.41-0.91). Furthermore, the median PFS of patients who underwent upfront surgery was 39.3 months in the olaparib/bevacizumab arm and 22.1 months in the placebo/bevacizumab arm (HR=0.47, 95% CI 0.29-0.75). The study showed that the PFS benefit was greater in cases where optimal debulking was performed, particularly in the upfront setting.

PRIMA/ENGOT-OV26 trial (42) evaluated niraparib for up to 3 years in patients with disease at high risk of treatment failure. In this trial, 258 (67%) patients who received NACT and then interval debulking surgery were included. The results of sub-analysis are expected.

VELIA/GOG-3005 trial  (43) evaluated PARP inhibition from the start of systemic treatment, concomitantly with chemotherapy, as well as in the maintenance setting, with veliparib administered for up to 2 years in the concomitant arm. NACT was administered to 134 patients, fifty-six of whom received veliparib and seventy-eight received placebo. The results of the sub-analysis are also awaited.

Trials evaluating PARP inhibitors in the front-line setting have several limitations (44). The results of SOLO-1 do not inform on patients with non-*BRCA*-mutated tumours. In addition, the trial lacked bevacizumab-containing therapy and prior use of bevacizumab was not permitted (40). Results from PAOLA-1/ENGOT-OV25 are also not applicable to patients considered ineligible for bevacizumab (41), while in PRIMA/ENGOT-OV26 the exclusion of patients with no visible residual disease after PDS, which is the goal of cytoreductive surgery, also limits the applicability of the results to a significant number of patients in routine oncology practice (42). Finally, the contribution of veliparib during the concomitant chemotherapy phase, in VELIA/GOG-3005 trial, is difficult to be defined in the absence of a fourth arm evaluating veliparib given only as maintenance therapy (43). The impact of the aforementioned limitations of NACT on clinical outcomes is yet to be determined. Selected current clinical trials investigating the role of PARP inhibitors in the neoadjuvant setting are shown in **Table 4**.

**Table 4:** Selected current clinical trials investigating the role of PARP inhibitors in the neoadjuvant setting.

Study Identifier	Type of Study	Study Population	Primary endpoint (s)	Regimen/Treatment Arms
NCT04598321	Phase I	Patients with ovarian, primary peritoneal or fallopian tube cancer (FIGO stage II-IV) and documented mutation in *BRCA1* or *BRCA2*	Proportion of patients completing the planned 9 weeks of treatment without disease progression	NAT^a^ with Talazoparib 1 mg orally daily for three cycles (defined as a 21-day period) followed by IDS and standard adjuvant therapy with Carboplatin and Paclitaxel and maintenance with Talazoparib
NCT03943173	Phase I	Patients with high grade ovarian, primary peritoneal or fallopian tube cancer (FIGO stage IIIC-IV) with documented BRCA pathway mutations	Feasibility	NAT with Olaparib on days 1-28. Treatment repeats every 28 days for up to 2 cycles in the absence of disease progression or unacceptable toxicity. After NAT, patients either undergo surgery then receive standard chemotherapy for up to 4 cycles or receive standard chemotherapy for up to 4 cycles then undergo surgery at the discretion of the treating physician
NCT04507841	Phase II	Patients with ovarian, primary peritoneal or fallopian tube cancer (FIGO stage III or IV)	R0 resection rate and ORR^b^ after NAT	NAT with niraparib 200 mg, once a day Time frame: 3-month
NCT04284852 NEOPRIMA	Phase II	Patients with ovarian, primary peritoneal or fallopian tube cancer (FIGO stage III-IV)	PFS^c^ rate at 18 months	3 - 4 cycles of NAT containing either Carboplatin or Cisplatin, then IDS^d^ with or without HIPEC, and 3-6 more cycles of adjuvant chemotherapy followed by Niraparib 200 or 300 mg daily up to 18 moths
NCT02489006 NEO	Phase II	Patients with ovarian, primary peritoneal or fallopian tube cancer	Difference in levels of PAR or PARP-1 before and after study treatment	Olaparib 300 mg orally twice per day for 6 weeks, followed by IDS, then followed by Platinum-based chemotherapy and Olaparib maintenance, after chemotherapy
NCT03393884 OVATION 2	Phase I Randomized Phase II	Patients with ovarian, primary peritoneal or fallopian tube cancer (FIGO stage III-IV)	PFS	*Arm A (Comparator)* NAT with 6 cycles of Paclitaxel 175 mg/m^2^ plus Carboplatin AUC 6 *Arm B (Experimental)* NAT as previously described with GEN-1^e^ 100 mg/m^2^ IP^f^ on days 8 and 15 of the first NAT cycle and then on days 1, 8, and 15 of the subsequent NAT cycles for a total of 17 doses.

a Neoadjuvant therapy.

b objective response rate.

c progression free survival.

d interval debulking surgery.

e a gene-based immunotherapy, comprising a human IL-12 gene expression plasmid and a synthetic plasmid delivery system, delivered intraperitoneally to produce local and persistent levels of IL-12.

f intraperitoneally.

Another promising field focuses on the possible synergistic effect of the concurrent use of PARPi and NACT. In a phase 1b trial, olaparib in combination with weekly paclitaxel and carboplatin in patients with relapsed EOC was shown to be effective and with acceptable toxicity, especially in patients with germline *BRCA* pathogenic variants (45). In this context, the NUVOLA (Neoadjuvant Chemotherapy in Unresectable Ovarian Cancer with OLAparib and Weekly Carboplatin Plus Paclitaxel), a phase II multicenter trial, was designed (46). The study primary endpoint is the pathological complete response rate after 3 cycles of NACT combined with olaparib in patients with high-grade serous ovarian cancer (HGSOC) carrying *BRCA* pathogenic variants. The estimated study completion date is December 2022.

## The role of immunotherapy

Cancer immunotherapy is a significant breakthrough in the treatment of cancer. These therapies, having less off-target effects, compared to standard chemotherapy, are implementing the immune system’s machinery to attack and ultimately kill cancer cells.

There are many studies where the presence of tumour infiltrating lymphocytes (TILs) is associated with a better survival outcome across diverse patients’ cohorts. A meta-analysis of 10 studies evaluating the prognostic value of TILs on survival among patients with ovarian cancer, demonstrated that intraepithelial CD8 and CD3 TIL had a prognostic role, with CD8 TIL being a more robust prognostic factor, regardless of tumor subtype, grade, or disease stage (47). Considering that phase-specific chemotherapeutic drugs such as taxanes, topotecan or pegylated liposomal doxorubicin, but not time-specific drugs like gemcitabine, were found to have a positive interaction with the activation of the immune system in mouse models, we can assume that patients with a high percentage of intraepithelial TILs may benefit from specific chemotherapy regimens (47, 48). With the evidence of the immunomodulatory activity of chemotherapeutic agents and the emerging immunotherapeutic strategies, the role of NACT in ovarian cancer acquires great interest.

Böhm et al. (49), investigated the effect of NACT on immune activation in stage III/IV tubo-ovarian HGSOC and its association to response to treatment. The investigators demonstrated that patients with a good response to NACT had reduced T-cell infiltration and more pronounced T-cell activation compared to poor responders. NACT also induced activation of CD4^+^ T cells, CD8^+^ T cells and CD45RO^+^ memory cells in omental metastases. Importantly, the levels of the immune-checkpoint molecules PD-1 and CTLA4 on CD4^+^ and CD8^+^ T cells remained high after NACT. In addition, a significant increase in the levels of PD-L1 on tumor infiltrating immune cells was reported. Finally, the systemic levels of three key inflammatory cytokines, IL6, IL8 and TNF were significantly raised after NACT.

Another group demonstrated that stromal TILs (sTILs) levels were prognostic at diagnosis and remained prognostic post-NACT (50). An overall increase in median stromal sTILs density from 20% to 30% was seen after NACT in patients with epithelial ovarian cancer. Especially, post-NACT, sTIL density had a predictive role for platinum-free interval (PFI), with patients with a PFI >6 months having significantly higher post-NACT sTIL density. Also, 33% of patients showed increasing intraepithelial TILs (ieTILs) density following NACT. In addition, the proportion of tumours with PD-L1-positive immune cells was 30% (15/50) pre-NACT and 53% (27/51) post-NACT (*p*=0.026). On multivariate analysis, only high sTILs both pre- and post-NACT were independent prognostic factors for PFS (HR=0.49, *p*=0.02 and HR=0.60, *p*=0.05, respectively). The remarkably interesting point in this study was that NACT had a significant effect on the tumour microenvironment (TME), with more than half of patients showing increased lymphocytic infiltration and upregulation of PD-L1 expression post-chemotherapy. Consequently, PD-1/PD-L1 blockade as maintenance treatment, post-NACT, could benefit patients with a lymphocyte-predominant, PD-L1 positive TME.

IMagyn050/GOG 3015/ENGOT-OV39 (NCT03038100) (51) was a double blind, placebo controlled, multicentre phase III trial, which enrolled patients with newly diagnosed stage III/IV ovarian cancer who underwent either PDS with gross residual disease or NACT and IDS. Of 1301 enrolled patients, approximately 25% received NACT, but although the combination was generally well tolerated with manageable adverse events, atezolizumab did not significantly improve PFS in the intention-to-treat or PD-L1-positive population.

Preliminary results of the AdoRN trial were presented at the 52^nd^ Annual Meeting on Women’s Cancer (52). The investigators evaluated atezolizumab in combination with NACT (paclitaxel 80 mg/m^2^ D1/8/15 + carboplatin AUC 6 D1 + atezolizumab 1200 mg D1, every 3 weeks for 3 cycles) followed by IDS for patients with newly diagnosed advanced-stage epithelial ovarian cancer. NACT with atezolizumab succeeded optimal cytoreduction in 86% of patients [R0 8 (53%), R1 (33%)]. Tumor and blood biomarkers results, which are expected, could identify potential candidates who may benefit from atezolizumab front-line therapy. Immunotherapy and NACT seem to have a synergistic role. The results of current clinical studies evaluating their combination are eagerly awaited. Selected current clinical trials investigating the role of immunotherapy in the neoadjuvant setting are shown in **Table 5**.

**Table 5:** Selected current clinical trials investigating the role of immunotherapy in the neoadjuvant setting.

Study Identifier	Type of Study	Study Population	Primary endpoint (s)	Regimen/Treatment Arms
NCT04163094	Phase I	Patients with epithelial ovarian cancer who are intended to be treated with NAT^a^	Change from baseline W_ova1 vaccine^b^ antigen-specific T cells in the peripheral blood	NAT with 3 cycles of Carboplatin + Paclitaxel, followed by IDS^c^, then followed by 3 additional cycles of chemotherapy. Patients will be vaccinated prior and during neoadjuvant chemotherapy with the W_ova1 vaccine.
NCT03126812	Phase I/II	Patients with ovarian, primary peritoneal or fallopian tube cancer (FIGO stage IV)	Number of T-cells in peripheral blood and tissue samples	NAT with Carboplatin AUC 6 (q 3 weeks) + weekly Paclitaxel 80 mg/m^2^ and Pembrolizumab 200 mg starting cycle 2 followed by IDS (Time Frame: at week 12)
NCT03899610 KGOG3046 TRU-D	Phase II	Patients with FIGO stage IIIC-IV ovarian cancer	PFS^d^	NAT with Paclitaxel 175 mg/m^2^ and Carboplatin AUC 5-6 + Durvalumab 1500 mg + Tremelimumab 75 mg (q 3 weeks for 3 cycles), followed by IDS, then followed by Chemotherapy + Durvalumab 1120 mg (q 3 weeks for 3 cycles) and maintenance with Durvalumab (9 cycles)
NCT02834975	Phase II	Patients with ovarian, primary peritoneal or fallopian tube cancer (FIGO stage III-IV)	pRR^e^	NAT with Paclitaxel 175 mg/m^2^ plus Carboplatin AUC 6 and Pembrolizumab 200 mg q 3 weeks
NCT04815408	Randomized Phase II	Patients with ovarian, primary peritoneal or fallopian tube cancer (FIGO stage IIIC-IV)	PFS	*Arm A (Comparator)* NAT with albumin-bound Paclitaxel 260 mg/m^2^ + Carboplatin AUC 5 (q3 weeks for 3 cycles), followed by IDS and HIPEC^f^, then followed by adjuvant treatment with albumin-bound Paclitaxel 260 mg/m^2^ + Carboplatin AUC 5 + Bevacizumab 7.5 mg/kg (q3 weeks for 3 cycles) *Arm B (Experimental)* The same treatment as in arm A with the addition of BGB-A317^g^ (200 mg q3 weeks for a total of 3 doses) to the neoadjuvant part of the treatment
NCT03393884 OVATION 2	Phase I Randomized Phase II	Patients with ovarian, primary peritoneal or fallopian tube cancer (FIGO stage III-IV)	PFS	*Arm A (Comparator)* NAT with 6 cycles of Paclitaxel 175 mg/m^2^ plus Carboplatin AUC 6 *Arm B (Experimental)* NAT as previously described with GEN-1^h^ 100 mg/m^2^ IP^i^ on days 8 and 15 of the first NAT cycle and then on days 1, 8, and 15 of the subsequent NAT cycles for a total of 17 doses.

a Neoadjuvant treatment.

b a vaccine consisting of mRNA encoding three tumor-associated antigens (TAAs) specific for ovarian cancer that are encapsulated in liposomes.

c interval debulking surgery.

d progression free survival.

e pathological response rate.

f hyperthermic intraperitoneal chemotherapy.

g tislelizumab (BGB-A317) is a humanized IgG4 anti–PD-1 monoclonal antibody specifically designed to minimize binding to FcγR on macrophages.

h a gene-based immunotherapy, comprising a human IL-12 gene expression plasmid and a synthetic plasmid delivery system, delivered intraperitoneally to produce local and persistent levels of IL-12.

i intraperitoneally.

## NACT and molecular profiling

### BRCA mutations and NACT

It is well known that about 15–25% of patients with EOC carry germline mutations in the *BRCA1* or *BRCA2* genes. The presence of these mutations is associated with a high susceptibility of HGSOC to cytotoxic treatment containing platinum, showing increased response rates, as well as improved five-year survival.

Specifically, studies have shown that patients with BRCA1/2 germline mutations demonstrate high platinum chemosensitivity to NACT. Gorodnova et al. showed that 34% patients with germline mutations had complete clinical response compared to 4% of wild type patients (53). Pathological complete response was observed in 46% of women with germline mutations in *BRCA1/2* compared to 24% of patients without such mutations. Interestingly, loss of heterozygosity of BRCA1 was observed in only 29% of post-NACT tumour tissues, whereas it was found in 82% of BRCA1 tumour tissues in chemonaive patients. However, loss of heterozygocity did not correlate with optimal cytoreduction, PFS or OS.

In another study, over-expression of several homologous recombination (HRR) genes appeared to be related with improved prognosis (54). RNA expression patterns were analysed from 96 fresh frozen HGSOC tumour samples, obtained either from patients who underwent PDS or NACT-IDS. In the NACT-IDS group, expression of RAD51 was independently associated with worse outcomes, while in the PDS group, overexpression of three genes (NBN, FANCF and RAD50) correlated with better prognosis (54). Therefore, the predictive and prognostic role of molecular alterations in HRR genes need to be further evaluated.

## Effect of NACT on tumor microenvironment

In recent years, the role of the TME in carcinogenesis and development of metastases has been extensively studied. Particularly, in ovarian cancer tumor promoting agents, including T lymphocytes and tumor-associated macrophages located in the ascites fluid, have been shown to promote infiltration and metastasis. It is also noteworthy that one of the main causes of disease progression and treatment failure is the inactivation of the immune response. The immune response through the TME is achieved with CD4^+^ T cells, CD8^+^ T cells and NK cells, which are directly suppressed not only by tumor cells but also by immunosuppressive Tregs, immature dendritic cells (DCs), tumor associated macrophages (TAMs) and myeloid derived suppressor cells (MDSCs).

Ovarian cancer is characterized by a unique TME. The role of resident host cells, as activated mesothelial cells in the peritoneal cavity and adipocytes of the omentum, is the main characteristic feature of the ovarian cancer TME (55). Cytokines and growth factors are released by macrophages, T cells, NK cells, adipocytes, mesothelial cells and fibroblasts, into the tumor microenvironment, thus playing an important role in tumor growth and progression, cancer dissemination and immune escape (56). Another important class of soluble cancer-promoting mediators in malignant effusions is the class of phospholipids, which include prostanoids, leukotrienes and eicositetranoic acids. More specifically, prostaglandin E2 promotes tumor progression by immune suppression and stimulation of angiogenesis (57). Extracellular microvesicles, which are released by both normal and tumor cells, mediate the transfer of lipids, proteins and nucleic acids and alter the function of the recipient cell. Extracellular microvesicles play a key role in immune evasion, tumor cell invasion and drug resistance (58, 59).

The effect of NACT on the microenvironment of ovarian cancer is an unexplored but critical field of study, as it could modify the response to other treatment options such as immunotherapy. Increased major histocompatibility complex (MHC) class I expression, T-cell infiltration, programmed death-ligand 1 (PD-L1) expression and a decrease of Treg cells in preclinical models after NACT were found on previous studies (60).

Jimenez-Sanchez et al. (61) studying HGSOC samples from two patient cohorts, a treatment naïve cohort consisting of 49 samples from 10 patients, and a paired pre- and post- NACT cohort from 40 patients, showed that TME of ovarian cancer is characterized by significant inter- and intra-patient heterogeneity. Investigators observed an increase of cytotoxic immunogenic activity after NACT in site-matched tumor samples but not in site-unmatched samples from the same patient. They also observed an increased number of T cell receptors (TCRs) before and after NACT, which implies that chemotherapy induced preexisting (neo)antigens in these patients. Another interesting observation was that NK cells were increased after NACT in site-matched samples, while no difference was observed in site-unmatched samples. There was no difference in cytotoxic cells or CD8+ between matched samples, therefore implying that NK cells mostly become active after NACT.

The induced immunogenicity after NACT poses the challenge for new combination therapies, such as targeting TME (including immune checkpoint inhibitors), inhibiting PARP or targeting angiogenesis.

## Effect of NACT on tumor molecular profile

Administration of chemotherapeutic regimens, either in the neo-adjuvant or adjuvant setting alters the mutational landscape and the gene expression in tumors and circulating-free DNA, respectively. Depending on the tumor type and the regimens used, different cellular pathways are affected. Due to the limited use of NACT in treating ovarian cancer, little is known on its actual effect on tumor genomic profile. Arend et al. (62), have made an attempt to record changes the expression profile in both the tumor and the plasma cell-free DNA of 14 patients that have been diagnosed with ovarian cancer and underwent NACT. Significant changes were observed in the expression of several genes, all of which operate in important molecular pathways and specifically, involve cell cycle regulation, ATM and GADD45 signaling. The burden of genetic variants identified in patient’s plasma was significantly reduced following NACT. On the contrary, most somatic variants identified remained unchanged following NACT.

The main indicators of the primary chemosensitivity based on the literature are pathological response score and biomarkers, genomic alterations, DNA scars, imaging, and circulating tumor markers. The tumor primary chemosensitivity affects the feasibility of R0 IDS after NACT, the efficacy of subsequent maintenance therapies with PARP inhibitors or bevacizumab, the risk of subsequent platinum-resistant relapse, and consequently PFS and OS. Provided that the completeness of the surgery may differ according to the primary chemosensitivity, we could assume that the maximum biological response (succeeded by systemic treatment) and the achievement of maximum debulking will maximize the OS (63).

## Conclusions

Despite novel therapeutic approaches, advanced ovarian cancer remains a disease with high mortality rate. In order to achieve a higher rate of complete cytoreduction, NACT and interval debulking were studied in different trials. NACT is associated with increased rates of optimal cytoreduction and decreased rates of peri-operative morbidity compared to PDS in patients with advanced ovarian cancer. In addition, no difference in primary survival outcomes between PDS and NACT has been determined. Therefore, NACT is recommended for the treatment of patients with FIGO stage IV, as well as for patients with stage III disease, where upfront optimal debulking cannot be achieved or surgery is contraindicated due to comorbidities. The role of additional therapeutic agents in improving clinical outcomes in patients receiving NACT is under evaluation. The evaluation of molecular biomarkers, including TME and genomic heterogeneity of HGSOC reveal novel therapeutic possibilities.

## Author Contributions

AN, concept, collecting data, and writing. EF, FF, HG, and AP, writing. CP, concept and writing. All authors contributed to the article and approved the submitted version.

## Conflict of Interest

The authors declare that the research was conducted in the absence of any commercial or financial relationships that could be construed as a potential conflict of interest.

## Publisher’s Note

All claims expressed in this article are solely those of the authors and do not necessarily represent those of their affiliated organizations, or those of the publisher, the editors and the reviewers. Any product that may be evaluated in this article, or claim that may be made by its manufacturer, is not guaranteed or endorsed by the publisher.
